# Turn Up the Heat—Food and Clinical *Escherichia coli* Isolates Feature Two Transferrable Loci of Heat Resistance

**DOI:** 10.3389/fmicb.2017.00579

**Published:** 2017-04-07

**Authors:** Erik J. Boll, Roger Marti, Henrik Hasman, Søren Overballe-Petersen, Marc Stegger, Kim Ng, Susanne Knøchel, Karen A. Krogfelt, Joerg Hummerjohann, Carsten Struve

**Affiliations:** ^1^Department of Microbiology and Infection Control, Statens Serum InstitutCopenhagen, Denmark; ^2^Agroscope, Division of Food Microbial Systems, Microbiological Safety of Foods of Animal Origin GroupBern, Switzerland; ^3^Department of Food Science, University of CopenhagenCopenhagen, Denmark

**Keywords:** heat resistance, *E. coli*, transfer of heat resistance, food production, biofilms, clpK

## Abstract

Heat treatment is a widely used process to reduce bacterial loads in the food industry or to decontaminate surfaces, e.g., in hospital settings. However, there are situations where lower temperatures must be employed, for instance in case of food production such as raw milk cheese or for decontamination of medical devices such as thermo-labile flexible endoscopes. A recently identified locus of heat resistance (LHR) has been shown to be present in and confer heat resistance to a variety of *Enterobacteriaceae*, including *Escherichia coli* isolates from food production settings and clinical ESBL-producing *E. coli* isolates. Here, we describe the presence of two distinct LHR variants within a particularly heat resistant *E. coli* raw milk cheese isolate. We demonstrate for the first time in this species the presence of one of these LHRs on a plasmid, designated pFAM21805, also encoding type 3 fimbriae and three bacteriocins and corresponding self-immunity proteins. The plasmid was highly transferable to other *E. coli* strains, including Shiga-toxin-producing strains, and conferred LHR-dependent heat resistance as well as type 3 fimbriae-dependent biofilm formation capabilities. Selection for and acquisition of this “survival” plasmid by pathogenic organisms, e.g., in food production environments, may pose great concern and emphasizes the need to screen for the presence of LHR genes in isolates.

## Introduction

Heat-treatment is an efficient and widely used measure to reduce bacterial contamination. If the material to be decontaminated is heat-stable, autoclaving can be used for complete sterilization. However, there are often situations where this is not feasible, both in food production and in the medical field, and lower temperatures must be employed. This is the case for heat treatment of flexible endoscopes (where thermochemical treatment below 60°C is being used; Jørgensen et al., 2016) or in case of thermization of raw milk for the production of specific cheeses (57–68°C for 15 s or more; Peng et al., 2013a), where protection of certain enzymes is aimed for. These treatments are generally sufficient to reduce the vegetative bacterial load to safe levels, unless the contaminating bacteria are especially heat-resistant.

Exposure of bacteria to severe heat stress results in massive misfolding and aggregation of proteins, and the potential toxic effects of these aggregates, coupled with net loss of active proteins, may cause cell death. Bacteria have evolved elaborate strategies to counteract these effects. Cytosolic chaperone systems with protein folding capacities such as DnaKJE and GroESL, as well as ATP-dependent proteolytic systems such as the Clp ATPases are ubiquitous in bacterial species (Hecker et al., 1996; Frees et al., 2014; Li and Gänzle, 2016). However, we recently identified a novel Clp ATpase, ClpK, uniquely found in Gram-negative bacteria, which also confers heat resistance (Bojer et al., 2010).

The *clpK* gene is located within a cluster termed the locus of heat resistance (LHR) comprising up to 16 open reading frames (ORFs) (Mercer et al., 2015). Located immediately upstream of *clpK* is a gene encoding a small heat shock protein, sHsp20c, which—like ClpK—has been shown to contribute to heat resistance (Lee et al., 2015). The remaining ORFs remain largely uncharacterized, but some are predicted to possess functional properties such as proteases or sodium/hydrogen transporters, implying that overall, the LHR may play a more generalized role in response to external stress (Mercer et al., 2015).

The LHR was originally described in *Klebsiella pneumoniae* and was found in roughly 1/3 of nosocomial *K. pneumoniae* isolates. The high prevalence of LHR in *K. pneumoniae* is likely due to its plasmid-located nature in this organism (Bojer et al., 2010). Notably, LHR-encoding plasmids often also carry multidrug-resistance genes and are transferable by conjugation to other *K. pneumoniae* isolates (Bojer et al., 2010, 2012). The LHR was since discovered in a *Cronobacter sakazakii* isolate, a pathogen associated with serious infections in neonates, which are thought to be linked to contaminated dried infant milk formula (Gajdosova et al., 2011).

Quite recently, a comparative genomic analysis of 29 *E. coli* strains identified a putatively chromosomally located ~14 kb region with >99% identity to the LHR clusters in *K. pneumoniae* and *C. sakazakii*. In contrast to the *K. pneumoniae* population examined, the LHR only occurred at a frequency of ~2% among the *E. coli* whole genomes and genome shotgun sequences published at that time (Mercer et al., 2015). We observed a similar frequency of LHR among extended-spectrum β-lactamase (ESBL)-producing *E. coli* isolates collected from Danish patients in 2008–2009 (Boll et al., 2016). This could suggest that the LHR does not provide significant general benefits to *E. coli*, or that transfer of LHR only rarely occurs in this organism. On the other hand, previous studies have investigated *E. coli* strains isolated from raw milk cheeses in Switzerland and shown that many of them exhibit increased heat resistance in milk at subpasteurization temperatures and during cheese ripening (Peng et al., 2012, 2013a,b). We recently screened a total of 256 of these *E. coli* raw milk cheese isolates for heat resistance markers *clpK* and an additional LHR marker gene, demonstrating that 93 (36.3%) of them were positive for both (while 24 and 9 isolates, respectively, were positive for one marker). We hypothesize that these increased numbers reflect a thermal selection pressure in this environment (Marti et al., 2016), due to the mild heat treatment employed during processing.

In this study, we focus on a raw milk cheese isolate, FAM21805, exhibiting a significantly increased level of heat resistance. We show that FAM21805 harbors two LHR variants, both of which confer heat resistance and both of which are transferrable by means of horizontal gene transfer. Moreover, one of the LHRs is located on a conjugative plasmid belonging to the IncFII group (pFAM21805) also harboring *mrkABCDF*, a locus encoding type 3 fimbriae (Burmølle et al., 2008). When transferred to *E. coli* K-12 MG1655, the plasmid increases both the heat resistance and biofilm formation properties of that strain. Finally, the plasmid is also transferable to strains of pathogenic *E. coli*. To the best of the authors' knowledge, this is the first description of a plasmid-borne LHR in *E. coli*.

## Materials and methods

### Bacterial strains and growth conditions

Bacterial strains used in this study are listed in Table 1. Bacteria were routinely cultured at 37°C on Luria-Bertani (LB) agar and in LB broth.

**Table 1:** ***E. coli*** **strains used in this study**.

**Strain**	**Misc.^a^**	**Origin**	**References**
FAM21805	Heat resistant; harbors LHR1 and LHR2	Swiss raw milk cheese	Peng et al., 2012, 2013a; Marti et al., 2016
FAM21805 ΔLHR1	Knock-out mutant, LHR1 (*orf2-orf16*) replaced with *kan^*r*^*		This study
FAM21805 ΔLHR2	Knock-out mutant, LHR2 (*orf2-orfF*) replaced with *kan^*r*^*		This study
FAM21805 ΔLHR1&2	Knock-out mutant, LHR1&2 replaced with *kan^*r*^*		This study
FAM21805 Δ*mrk*	Knock-out mutant, *mrkABCDF* replaced with *tet^*r*^*		This study
FAM21805 Δ*orfE*	Knock-out mutant, *orfE* replaced with *tet^*r*^*		This study
FAM21805 LHR1 *kan^*r*^*	LHR1 tagged with *kan^*r*^* without knock-out of known ORFs; donor of LHR1 in HGT experiments		This study
FAM21805 LHR2 *tet^*r*^*	LHR2 tagged with *tet^*r*^* without knock-out of known ORFs; donor of LHR2 in HGT experiments		This study
C604-10	Heat resistant, ESBL-producing	Human clinical isolate	Olesen et al., 2013; Boll et al., 2016
C604-10 LHR1 *kan^*r*^*	LHR1 tagged with *kan^*r*^* without knock-out of known ORFs; donor of LHR1 in HGT experiments		Boll et al., 2016
C604-10 LHR2 *tet^*r*^*	LHR2 tagged with *tet^*r*^* without knock-out of known ORFs; donor of LHR2 in HGT experiments		Boll et al., 2016
MG1655 *nal^*r*^ rif^*r*^*	Recipient in HGT experiments, laboratory strain	Commensal K-12 strain	Møller et al., 2003
MG1655 LHR2 (21805)	MG1655 transconjugant harboring LHR2 plasmid of FAM21805 tagged with *tet^*r*^* without knock-out of known ORFs		This study
MG1655 LHR1&2 (21805)	MG1655 transconjugant harboring LHR1&2 of FAM21805; LHR1 tagged with *kan^*r*^* without knock-out of known ORFs		This study
MG1655 LHR2 Δ*orfE* (21805)	MG1655 transconjugant harboring LHR2 plasmid of FAM21805 tagged by replacing a putative di-guanyl cyclase (*orfE*) with *tet^*r*^*		This study
MG1655 LHR2 Δ*mrk* (21805)	MG1655 transconjugant harboring LHR2 plasmid of FAM21805 tagged by replacing *mrkABCDF* with *tet^*r*^*		This study
MG1655 LHR1 (C604-10)	MG1655 transconjugant harboring LHR1 of C604-10; tagged with *kan^*r*^* without knock-out of known ORFs		This study
MG1655 LHR2 (C604-10)	MG1655 transconjugant harboring LHR2 of C604-10 tagged with *tet^*r*^* without knock-out of known ORFs		This study
FAM21843	Heat resistant; harbors LHR1	Swiss raw milk cheese	Peng et al., 2012, 2013a; Marti et al., 2016
FAM22873	*eaeA* (intimin) +, *stx1*+, serotype O26+	Swiss raw milk	This study
FAM22873 LHR2 (21805)	FAM22873 transconjugant harboring LHR2 plasmid of FAM21805 tagged with *tet^*r*^* without knock-out of known ORFs		This study
FAM22873 ΔLHR2 (21805)	FAM22873 transconjugant harboring LHR2 plasmid of FAM21805 tagged by replacement of LHR with *kan^*r*^*		This study
FAM23288	*stx2*+	Swiss raw milk	This study
FAM23288 LHR2 (21805)	FAM23288 transconjugant harboring LHR2 plasmid of FAM21805 tagged with *tet^*r*^* without knock-out of known ORFs		This study
FAM23288 ΔLHR2 (21805)	FAM23288 transconjugant harboring LHR2 plasmid of FAM21805 tagged by replacement of LHR with *kan^*r*^*		This study
55989	Enteroaggregative *E. coli* (EAEC) strain	Human clinical isolate	Bernier et al., 2002
55989 LHR2 (21805)	55989 transconjugant harboring LHR2 plasmid of FAM21805 tagged with *tet^*r*^* without knock-out of known ORFs		This study
55989 ΔLHR2 (21805)	55989 transconjugant harboring LHR2 plasmid of FAM21805 tagged by replacement of LHR with *kan^*r*^*		This study
C227-11 φcu	Shiga toxin-producing EAEC strain cured of the Stx2a-bearing phage	Human clinical isolate	Zangari et al., 2013
C227-11 φcu LHR2 (21805)	C227-11 φcu transconjugant harboring LHR2 plasmid of FAM21805 tagged with *tet^*r*^* without knock-out of known ORFs		This study
C227-11 φcu ΔLHR2 (21805)	C227-11 φcu transconjugant harboring LHR2 plasmid of FAM21805 tagged by replacement of LHR with *kan^*r*^*		This study.

a *kan^r^, kanamycin resistance cassette; tet^r^, tetracycline resistance cassette; nal^r^, nalidixic acid resistant; rif^r^, rifampicin resistant*.

### Screening PCRs used in this study

A total of 90 *E. coli* dairy isolates were screened for LHR marker genes *clpK1* and *clpK2*, and the *mrkABCDF* locus encoding type 3 fimbriae using the primers and annealing temperatures indicated in Table 2.

**Table 2:** **Primers and annealing temperatures used in this study**.

**Name**	**Sequence (5′-3′)**	**Target**	**Use**	**T_ann._ (°C)**	**References**
K12-R	ATCCTGCGCACCAATCAACAA	orf264 and the IS element inserted only in K-12 strains e.g., MG1655	Confirmation of transconjugant identity	54	Kuhnert et al., 1995
K12IS-L	CGCGATGGAAGATGCTCTGTA				
clpK1_F	TGCTGTTGTGCGACGACCATTACC	*clpK1* gene specific PCR	Screening of strains for presence of *clpK1*	64	Boll et al., 2016
clpK1_R	TTGCCGACCACCTTGCTGACCTGT				
clpK2_F	ACGATCACTATCGCCAACTG	*clpK2* gene specific PCR	Screening of strains for presence of *clpK2*	64	Boll et al., 2016
clpK2_R	AGTATTTATCCAGCTCGGGCGTG				
orfE_F	CGGTCGTTCTGGCAAAGGTG	putative di-guanyl-cyclase (dgc) of FAM21805 LHR2	PCR specific for *orfE*, a putative dgc, part of LHR2 plasmid	64	This study
orfE_R	CGTCCTGACGAAATCGCTCC				
mrkD_F	TCGAAGGGTCGCGCTTTACG	*mrkD* of the *mrkABCDF* locus	Screening of strains for presence *mrk* locus encoding type 3 fimbriae	60	This study
mrkF_R	CATGGTAGCGGTAGTGCTGGTGG				

### Phenotypic heat resistance screen

*E. coli* dairy isolates were screened for phenotypic heat resistance by incubation of overnight cultures LB Lennox broth (LB, 10 g/L tryptone, 5 g/L yeast extract, 5 g/L sodium chloride, pH 7.0) at 55°C for 30 min. Strains were diluted 1:10 into pre-heated LB broth and sampled at 0, 15, and 30 min. (duplicate plating). Strains showing a reduction in colony forming units (CFU) of less than one log_10_ after 30 min. were considered phenotypically heat resistant. When comparing wildtype strains with their LHR mutants or when testing LHR transconjugant strains, a fourth time point at 45 min incubation was added and the assay was performed at least in biological triplicate. Relative survival of a strain at a given time point was calculated by dividing the CFU/ml of that time point by the initial CFU/ml (time point 0).

### Genome sequencing

FAM21805 and FAM21843 genomic DNA was extracted with the GenElute™ Bacterial Genomic DNA Kit (Sigma-Aldrich, Buchs, Switzerland). Illumina sequencing was done in a 101-bp paired-end run (University of Bern), and *de novo* assembly was performed using CLCbio Genomic Workbench (v9.0.1). For all other strains, genomic DNA was extracted from isolates using a DNeasy Blood and Tissue Kit (QIAGEN, Copenhagen, Denmark). MiSeq libraries were made using the NexteraTMKit (Illumina) and sequencing was performed as 250-bp paired-end runs. Reads were assembled *de novo* using CLCbio Genomic Workbench (v9.5.2). The annotated sequence of LHR1_FAM21805_ has been deposited at GenBank under the accession KY646173. The Whole Genome Shotgun projects of FAM21805 and FAM21843 have been deposited at DDBJ/ENA/GenBank under the accessions MVEA00000000 and MVIN00000000, respectively.

### Plasmid sequencing

Plasmid DNA was sequenced both on a MiSeq instrument (Illumina) and on a MinION flow cell (Oxford Nanopore Technologies). The MiSeq library was made using the Nextera XT kit (Illumina) and sequencing was performed as a paired-end 250 bp run yielding 372,720 reads with an average length of 237 bp. MinION library with native barcode (NB01 from EXP-NDB002) was prepared using the R9 Genomic Sequencing kit (SQK-NSK007) and was sequenced on a FLO-MIN105 SpotON Mk1 flow cell according to the manufacturer's instructions. Fast5 read files were subjected to base calling via a two-direction (2D) workflow using ONT's Metrichor software yielding 12,327 passed read files. Mixed assembly was performed by combining MiSeq and MinION reads using the SPAdes (v3.9.0) assembler. Finally, CLCbio Genomic Workbench (v9.5.2) was used for end trimming of the assembled plasmid and for final error correction by mapping trimmed MiSeq reads against the plasmid contig obtained after the mixed SPAdes assembly. ORFs were predicted by RAST annotation (Aziz et al., 2008) and then manually curated. The sequence has been deposited at GenBank under the accession KY416992.

### Phylogenetic analysis

For phylogenetic analysis of the LHR, genomes containing homologous sequences with >80% coverage of LHR2 of raw milk cheese *E. coli* isolate FAM21805 were retrieved from NCBI. In addition, sequences from clinical ESBL-producing *E. coli* isolates C598-10 and C604-10 (Boll et al., 2016), beef *E. coli* isolates AW1.3, AW1.7, and GM16.6-6 (Mercer et al., 2015), raw milk cheese *E. coli* isolate FAM21843 (Peng et al., 2012, 2013a) as well as five clinical isolates from Statens Serum Institut were included. Single nucleotide polymorphisms (SNPs) were identified after alignment of all sequences to the LHR1_FAM21805_ reference using the NUCmer component (Kurtz et al., 2004) as implemented in the Northern Arizona SNP Pipeline (NASP) v1.0 (http://biorxiv.org/content/early/2016/01/25/037267). A total of 1,270 SNPs were identified from 45% of the 15 kb LHR1_FAM21805_ excluding any repetitive regions. The relatedness of the elements was inferred using the maximum-likelihood algorithm implemented in PhyML (http://www.atgc-montpellier.fr/phyml-sms/) with Smart Model Selection using the Bayesian Information Criterion with 100 bootstrap replicates using random starting trees.

### Modification of bacterial strains

LHR1 in FAM21805 was deleted by allelic exchange with a kanamycin resistance (*kan*^*r*^) encoding cassette, flanked by regions homologous to sequences at the beginning and end of the loci, as previously described (Bojer et al., 2010). The *kan*^*r*^ cassette was then removed from FAM21805ΔLHR1 using pCP20. The LHR2 locus was deleted in FAM21805 and FAM21805ΔLHR1 by allelic exchange with *kan*^*r*^ flanked by regions homologous to sequences at the beginning and end of LHR2. Using the same technique, the *mrkABCDF* cluster and *orfE* (encoding a putative digyanylate cyclase within LHR2) in FAM21805 were deleted by allelic exchange with a tetracycline resistance (*tet*^*r*^)-encoding cassette. Finally, *kan*^*r*^ or *tet*^*r*^ were introduced immediately downstream of the last ORFs of LHR1 and LHR2 without disrupting the flanking mobile elements (Table 1).

### Horizontal gene transfer experiments

Horizontal gene transfer of loci of heat resistance was assessed in plate matings as previously described (Marti et al., 2016). In short, 500 μl overnight culture of donor and recipient strains were mixed and centrifuged (12,000 × g, 2 min), the supernatant completely removed and resuspended in 50 μl NA (8 g/L NaCl, 1 g/L peptone) solution and spotted onto LB Agar plates. The rifampicin (RIF) and nalidixic acid (NAL) resistant *E. coli* K-12 MG1655 rif^*r*^ nal^r^ (Møller et al., 2003) was used as recipient. The donors were various heat resistant strains with LHRs tagged with either *tet*^r^ or *kan*^*r*^ cassettes (Table 1). After incubation at 37°C for 24 h (if not stated otherwise), the cells were scraped off, suspended in 3 ml NA and plated onto LB plates selective for donors (LB_TET_ or LB_KAN_), recipients (LB_NAL/RIF_), and transconjugants (LB_NAL/RIF/TET_ or LB_KAN/NAL/RIF_). After overnight incubation at 37°C, CFU were counted and transconjugant frequencies per recipient calculated. The antibiotics were used in the following concentrations: Kanamycin: 50 μg/ml, nalidixic acid: 30 μg/ml, tetracycline: 15 μg/ml, and rifampicin: 100 μg/ml (Sigma-Aldrich, Buchs, Switzerland). The assay was carried out in biological triplicate except where frequencies were too low for quantitative evaluation. Three presumptive K-12 MG1655 transconjugants per replicate were confirmed by screening with MG1655-specific primers and various combinations of specific PCRs for *clpK1, clpK2, orfE*, or *mrkD* (Table 2).

### Biolog phenotype MicroArrays

Stress responses of *E. coli* FAM21805 wild-type and its LHR mutants, as well as *E. coli* K-12 transconjugants, were assessed using the Phenotype MicroArrays (PM) 9 and 10 of the Biolog system screening for growth depending on osmolytes and pH (BIOLOG, Inc., Hayward, CA, USA). Strains were prepared and plates inoculated as follows: Strains from glycerol stocks were re-activated by two overnight incubations at 37°C on blood agar plates (Columbia agar + 5% sheep blood, bioMérieux, Geneva, Switzerland). The second plate was less than 24 h old when used for inoculation of the PM plates. Inoculating fluids (IF) were prepared by addition of 25 ml sterile water to 125 ml IF-0a and addition of 1.5 ml dye mix A and 23.5 ml water to 125 ml IF-10b. Cells were inoculated into IF-0 to a transmittance of 42 ± 2% using a sterile cotton swab and diluted (1:5) into fresh IF-0 resulting in a final transmittance of 85 ± 2%. This suspension was diluted 1:200 into IF-10b + dye mix A and used to inoculate PM plates (100 μl per well). Metabolic activity was assessed at 37°C for 72 h under standard atmosphere. PM plates, IF-0 and IF-10b were obtained from Endotell AG, Allschwil, Switzerland.

### Hydrogen peroxide growth challenge

In a first step, overnight cultures were diluted 1:100 into fresh tryptic soy broth (TSB, Oxoid, Pratteln, Switzerland). This was followed by a second 1:10 dilution into pre-warmed TSB containing hydrogen peroxide (H_2_O_2_) resulting in 1 ml aliquots with final concentrations of 0, 0.5, 1, 2, 5, 7.5, 10, 12.5, and 15 mM H_2_O_2_. For each concentration, a quadruplicate of 200 μl per well was added to a 96-well plate and incubated at 37°C for 24 h in a microplate reader (model: ELx808, BioTek, Luzern, Switzerland). Optical density at λ = 600 nm (OD_600_) was measured in intervals of 30 min. The plates were only shaken for 5 s prior to each measurement on the fast setting. The experiment was carried out in biological duplicate.

### Hydrogen peroxide inactivation assay

Overnight cultures were diluted 1:10 into 0.9% NaCl solution pre-warmed to 37°C with a final H_2_O_2_ concentration of 50 mM and statically incubated at 37°C. Following 0, 15, and 30 min of incubation, 50 μl samples were taken (mixed by pipetting), and immediately diluted 1:10 into 450 μl 0.9% NaCl solution. This immediately reduced the H_2_O_2_ concentration and stopped further reduction of CFU. Once all samples were taken, further dilutions were spotted in triplicate on TSA (Oxoid, Pratteln, Switzerland) and incubated overnight at 37°C.

### Crystal violet (CV) biofilm formation assay

Overnight cultures of strains grown in LB broth were diluted 1:100 in minimal media with 0.5% casamino acids as carbon source (ABTCAA, Reisner et al., 2006) and 150 μl were added per well (eight wells per strain and biological replicate) in 96-well plates (untreated PS surface, CytoOne, StarLab, Hamburg, Germany). Plates were wrapped in plastic bags and partially closed to reduce evaporation of media and incubated at 12, 28, and 37°C for 48 h. After incubation, the plates were emptied by throwing out and removing residual liquid by touching the inverted plate on paper tissue. Plates were then washed three times with 200 μl NA per well and the biofilms subsequently stained with 200 μl 0.1% crystal violet (CV) solution (Sigma-Aldrich, Buchs, Switzerland) per well for 20 min. Staining was followed by three washes with ddH_2_O and biofilms were dissolved in 200 μl 96% ethanol per well. Biofilm formation was assessed by measurement of OD_600_ values, which are reported as average and standard deviation of three biological replicates of OD_600_ of strains minus OD_600_ of the media control.

### Plasmid profiling

For plasmid size determination, plasmid preparation was carried out using a modified version of the protocol by Kado and Liu (1981) as previously described (Schjørring et al., 2008). As a plasmid marker, *E. coli* strain 39R861 was used, containing four plasmids of 147, 63, 36, and 7 kb (Threlfall et al., 1986).

### Statistical analysis

Statistical analysis of data (*t*-tests, Mann-Whitney rank sum tests and 1-way ANOVA) was performed using SigmaPlot 13.0 (Systat Software, San Jose, CA) as indicated in the main text and legends (α = 0.05).

## Results

### Two distinct heat resistance clusters are present in *E. coli* raw milk cheese isolates

We have recently described the presence of two loci of heat resistance (LHR1 and LHR2) in a clinical ESBL-producing *E. coli* isolate (C604-10), both of which contributed to a highly heat resistant phenotype (Boll et al., 2016). In a separate study, we reported a remarkably high frequency of *clpK*-positive *E. coli* raw milk or raw milk cheese isolates (93 out of 256) (Marti et al., 2016). Here, we examined a subset of these isolates (both *clpK*-positive and—negative) for the potential presence of both LHRs with PCRs specific for *clpK1* and *clpK2*, the marker genes of LHR1 and LHR2, respectively. Of the 90 tested isolates, 23 contained both, 26 were positive for *clpK1* only and one only for *clpK2*. Next, we correlated PCR results and phenotypical heat resistance to determine the predictive value of the PCRs. All of the 49 *clpK1* positive strains were heat resistant and only one out of 50 heat resistant strains was *clpK1* negative. Thus, there was a strong correlation of heat resistance with *clpK1*. Every strain positive for *clpK2* (Stahlhut et al., 2013) tested phenotypically heat resistant, but only 24 of 50 heat resistant strains were *clpK2* positive. It is important to note that the *clpK1* negative, yet heat resistant strain was *clpK2* positive. Thus, the combination of these two PCRs resulted in perfect prediction of phenotypic heat resistance in this set of strains (double PCR negative strains all tested heat sensitive).

Strains testing positive for both *clpK1* and *clpK2* by PCR showed significantly increased survival in our phenotypic heat resistance screening assay compared to the *clpK1* single positive strains, which in turn were significantly more resistant than double negative strains. The average relative survival after 30 min at 55°C was 4.64 ± 6.61 × 10^−3^ for double negatives (*n* = 40), 3.27 ± 1.75 × 10^−1^ for *clpK1* single positives (*n* = 26), and 5.39 ± 1.96 × 10^−1^ for *clpK1* and *2* double positive strains (*n* = 23). The differences between groups are statistically significant (*p*-values < 0.001 for all pairwise comparisons, Mann-Whitney Rank Sum Test). For further characterization of the two different LHR clusters in our collection of raw milk and raw milk cheese isolates, we focused our attention on isolate FAM21805, which harbors both *clpK* gene variants. This strain has previously been shown to exhibit an increased degree of heat resistance compared to strains harboring a single LHR in milk at sub-pasteurization temperatures (Peng et al., 2013a).

### Characterization of a plasmid-encoded LHR in raw milk cheese isolate FAM21805

**Figure 1:**
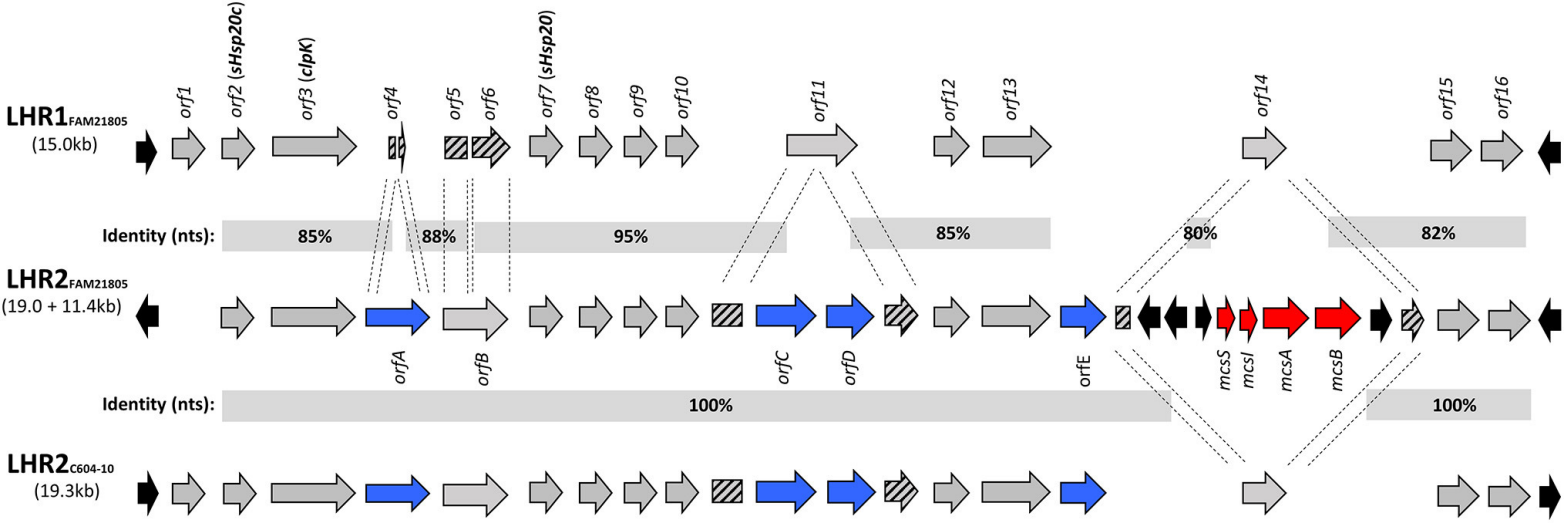
**Comparison of loci of heat resistance in FAM21805 (LHR1 and -2) and C604-10 (LHR2)**. ORFs present in all three LHRs are marked in gray (except for *orf1* which is absent in LHR2_FAM21805_), ORFs unique to LHR2 are marked in blue, partially disrupted ORFs are marked with stripes, and mobile elements are marked in black. Regional nucleotide identities (%) are depicted. A 11.4 kb region containing the *mcsSIAB* cluster (Microcin S system, marked in red) has been inserted into LHR2_FAM21805_. For additional information on the ORFs, see Supplementary Table 1.

Whole genome sequencing (Illumina) analysis revealed that raw milk cheese isolate FAM21805 harbored an LHR (here designated LHR1) ~15 kb in size with a G+C content of 62% and highly similar (98–99%) to the one previously described in four heat resistant *E. coli* isolated from beef (Mercer et al., 2015). It contained fourteen putative ORFs and was flanked by mobile elements (Figure 1). We moreover detected additional homologs to several of these ORFs, strongly implying the presence of a second LHR in this isolate. However, *de novo* assembly failed to demonstrate the location of these homologs within a single genetic locus.

Since in *K. pneumoniae*, the LHR is thought to predominantly be located on plasmids, we sought to determine whether this was also the case for this putative additional LHR in *E. coli* isolate FAM21805. Gel electrophoresis of purified plasmids revealed that FAM21805 harbored two large plasmids of slightly different sizes around 110–120 kb (Supplementary Figure 1). We therefore introduced a kanamycin resistance encoding gene (*kan*^*r*^) within the *clpK2* gene, purified the plasmids from the resulting FAM21805 Δ*clpK2* strain and transformed the plasmids into laboratory *E. coli* strain NEB-10β. Plating on kanamycin-containing plates yielded several colonies, and plasmid profiling from one of these demonstrated the presence of a single plasmid in NEB-10β corresponding in size to the lower-size plasmid in isolate FAM21805 (Supplementary Figure 1), strongly suggesting that the *kan*^*r*^–disrupted *clpK2* gene was in fact located on that plasmid.

**Figure 2:**
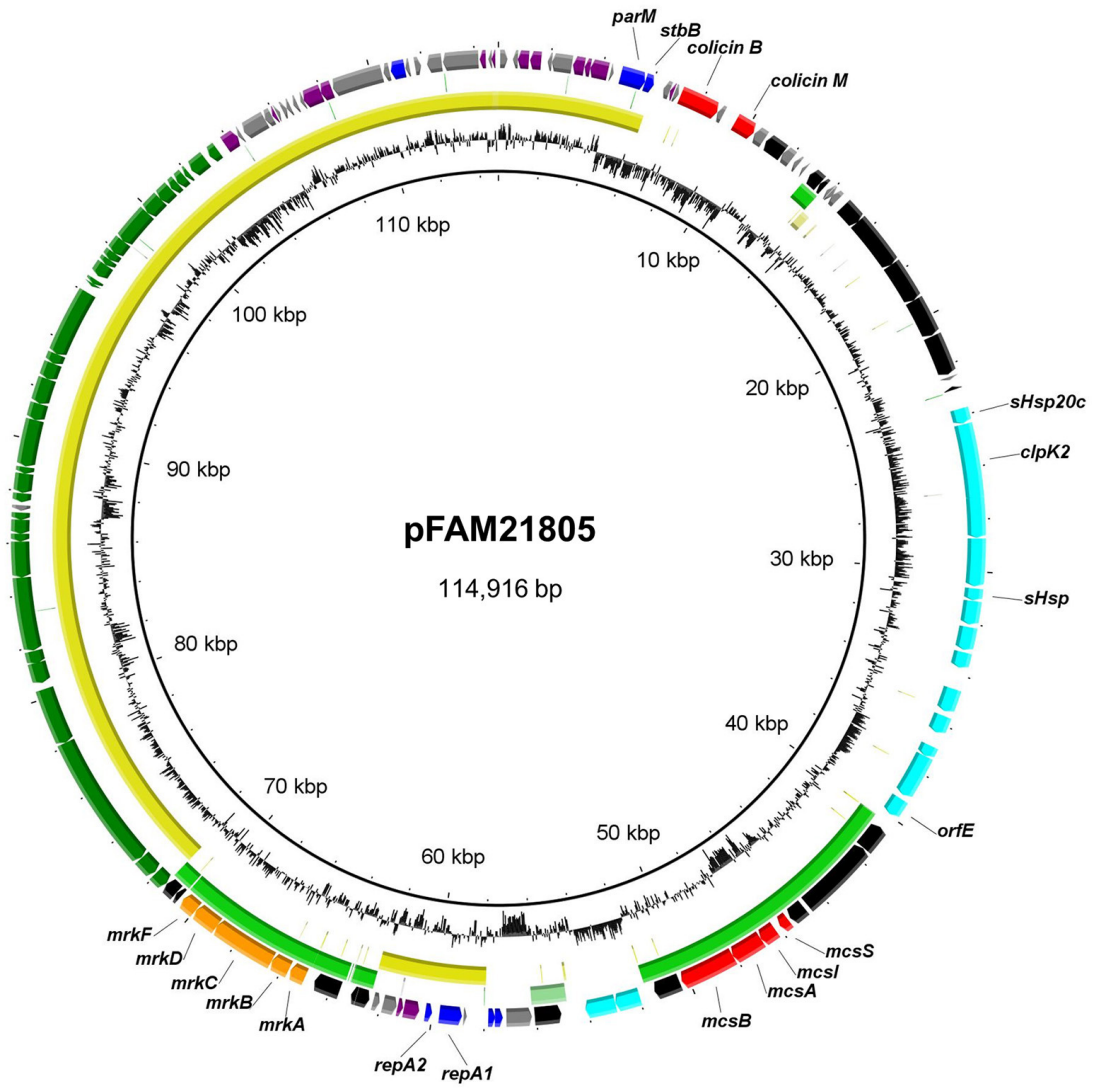
**Circular representation of pFAM21805 and BLASTn comparison of plasmids with shared regions**. The outer ring shows predicted ORFs. Colors represent different putative functions: Gray, hypothetical proteins; blue, plasmid replication and maintenance; red, bacteriocins and accessory genes; light blue, LHR2 genes; orange, adhesins; dark blue, regulatory genes; purple, miscellaneous; and black, mobile elements. Within circles two (pCPO13026, yellow) and three (pSYM1, green), the darkest color indicates nucleotide identity exceeding 90%, whereas the lightest color represents identity exceeding 80%. Innermost circle, G+C content. The circular map was generated using BRIG.

Using MinIon Nanopore R9 technology, we sequenced the *kan*^*r*^-tagged plasmid from the transformed NEB-10β strain. We then manually replaced the *kan*^*r*^–disrupted *clpK2* gene with the intact *clpK2* from Illumina sequencing within the complete closed sequence, thus reconstructing the original plasmid sequence. The resulting plasmid, titled pFAM21805, is 114,916 bp in length and has an average G+C content of 53.6% (Figure 2). RAST annotation predicted a total of 136 ORFs, 99 of which were functionally assigned. A single RepII replicon was identified with the F plasmid type FAB formula F96:A-:B-.

The plasmid sequence confirmed the presence of a single LHR locus (LHR2_FAM21805_) ~19.0 kb in length and containing 15 putative functional ORFs (Figure 1 and Supplementary Table 1). It had a G+C content similar to LHR1 (61%) and was flanked by various mobile elements. Homologs to key elements of LHR1 were also present in LHR2_FAM21805_, including: (1) the Clp ATPase ClpK (*orf3*); (2) the two small heat shock proteins, sHsp20c (*orf2*) and sHsp20 (*orf7*); (3) a putative sodium/hydrogen exchanger with a Kef-type membrane component (*orf13*); (4) a putative zinc-dependent protease (*orf15*); and (5) a putative 2-alkenal reductase with a trypsin-like protease domain (*orf16*). Overall, 75% of LHR2_FAM21805_ was present in LHR1_FAM21805_ with a corresponding average identity of 88% (Figure 1).

The previously found LHR2 in ESBL-producing *E. coli* isolate C604-10 was highly identical to LHR2_FAM21805_ (Boll et al., 2016). However, whereas LHR2_C604−10_ (and LHR1_FAM21805_) was initiated by an ORF encoding a protein with a putative helix-turn-helix (HTH) motif capable of binding DNA (*orf1*), this ORF was absent in LHR2_FAM21805_. Moreover, a region spanning 11.4 kb with a G+C content of 50% had been inserted into *orf14* of LHR2_FAM21805_ (Figures 1, 2). This region comprised the *mcsSIAB* gene cluster encoding an antibacterial peptide Microcin S (MccS) along with a self-immunity protein and transport apparatus recently identified on plasmid pSYM1 in probiotic *E. coli* strain G3/10 (Zschüttig et al., 2012).

LHR2 of FAM21805 and C604-10 both contained four ORFs not found in LHR1: (1) a putative cardiolipin synthase (*orfA*); (2) a putative mechanosensitive ion channel (*orfC*) followed by a hypothetical protein of unknown function (*orfD*); and (3) an ORF containing a GGDEF domain characteristic of a putative di-guanyl cyclase (*orfE*) (Figure 1 and Supplementary Table 1). Interestingly, remnants of the N- and C-terminal parts of *orfA* were present in LHR1, indicating evolutionary partial loss of this ORF in LHR1. In addition, *orfC* and *orfD* were inserted in the middle of *orf11* encoding a hypothetical protein in LHR1, thereby disrupting this ORF. Finally, *orfE* appeared to have been inserted between *orf13* and *orf14* present in both LHRs (Figure 1).

In addition to LHR2 itself, pFAM21805 also contained the *mrkABCDF* cluster, encoding type 3 fimbriae, which mediate bacterial biofilm formation on abiotic surfaces such as plastic, and on biotic surfaces such as human epithelial cells. The *mrk* operon is present in nearly all *K. pneumoniae* isolates but is only rarely found in *E. coli* (Stahlhut et al., 2013). Notably, plasmid co-localization of the *mrk* operon and the *mcs* gene cluster was also observed in the probiotic G3/10 strain (Zschüttig et al., 2012). pFAM21805 also harbored a 32 kb *tra* region (24 *tra* genes, 8 *trb* genes, and *finO*), implying that the plasmid may be transferable by conjugation. Also present on the plasmid were genes associated with plasmid stability including *parM* and *stbB* as well as the genes encoding the antimicrobial compounds Colicin B and Colicin M (Figure 2). Thus, the plasmid likely confers both enhanced heat resistance, the ability to kill other *E. coli* and enhanced adhesive properties to FAM21805.

A large portion of pFAM21805, including the *tra* region, *repA* replicon and plasmid maintenance genes also comprised the backbone of plasmid pCPO13026 of Shiga Toxin-producing *E. coli* (STEC) strain 2009C-3133 isolated from a patient in New York in 2009 (Lindsey et al., 2015) (Figure 2).

### Presence of loci of heat resistance in other pathogenic species

**Figure 3:**
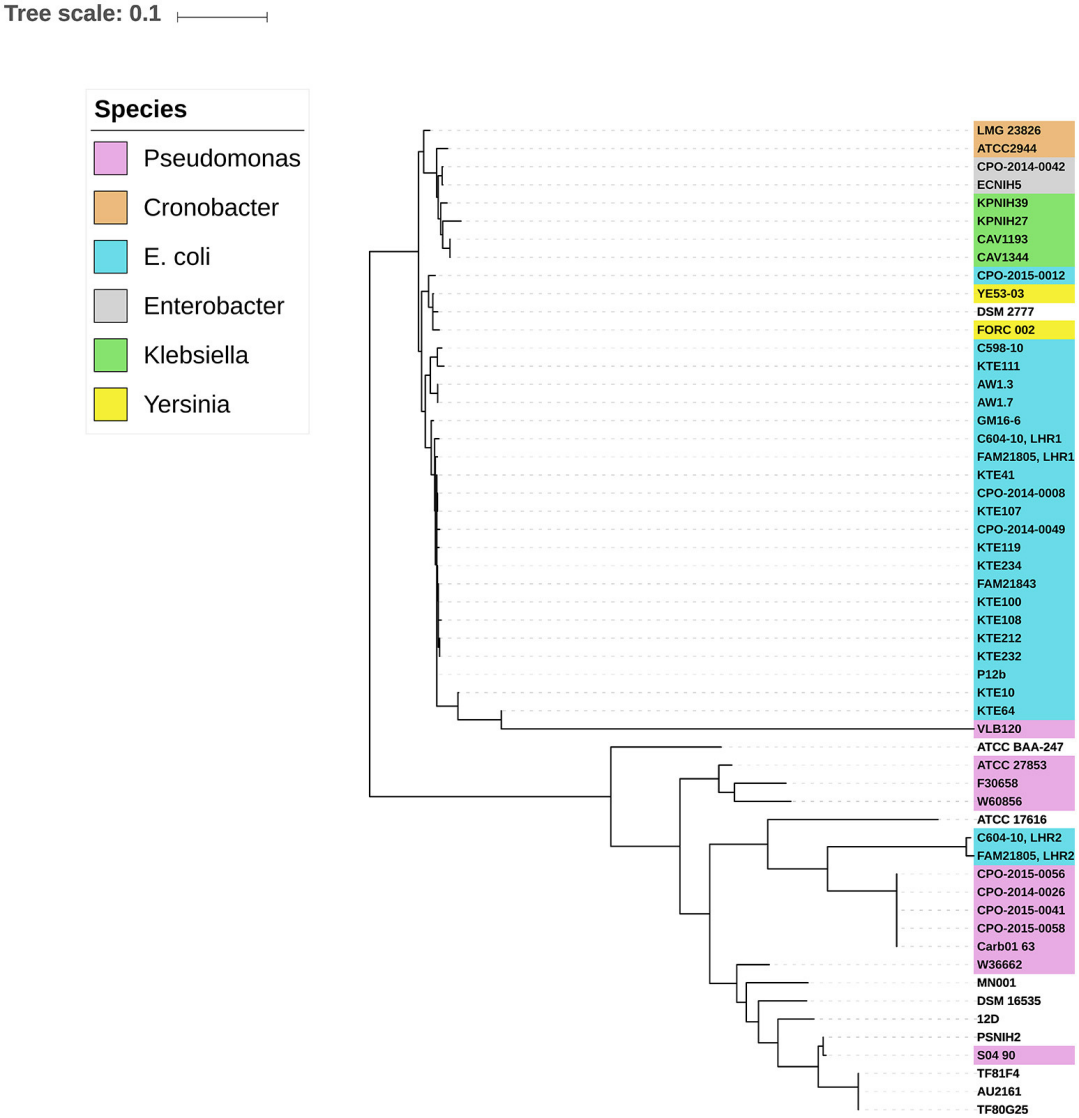
**Midpoint-rooted maximum-likelihood phylogenetic tree of aligned LHR sequences (>80% coverage of LHR1_FAM21805_) from different bacterial species**. Two distinct clusters representing predominantly *Enterobacteriaceae* and strains of *Pseudomonas*, respectively, are observed. LHR2 of FAM21805 and C604-10 cluster within the *Pseudomonas* group.

Previous studies have described the presence of LHR in several distinct pathogenic species, including *E. coli, K. pneumoniae, Enterobacter* species, and *Pseudomonas aeruginosa* (Bojer et al., 2010; Gajdosova et al., 2011; Lee et al., 2015; Mercer et al., 2015). Based on a BLAST search using the entire LHR, Mercer et al. recently observed two distinct phylogenetic groups harboring LHRs—one predominantly comprising *Enterobacteriaceae* and one primarily comprising *P. aeruginosa* (Mercer et al., 2015). We performed the same analysis using LHR2 from FAM21805 as input, which retrieved 27 sequences with more than 80% coverage. SNPs within the LHRs were identified and used to calculate a maximum-likelihood phylogenetic tree. As expected, LHR1_FAM21805_ and LHR1_C604−10_ both clustered tightly with the other *E. coli* LHR1s within the *Enterobacteriaceae* group (Figure 3). Not surprisingly, LHR2_FAM21805_ and LHR2_C604−10_ both clustered tightly together. Remarkably, however, they were located within the *Pseudomonas* group of the phylogenetic tree. These findings demonstrate the ability of *E. coli* to acquire LHRs from both phylogenetic groups.

### Both the LHR1 and LHR2 confer heat resistance and are transferable by conjugation

**Figure 4:**
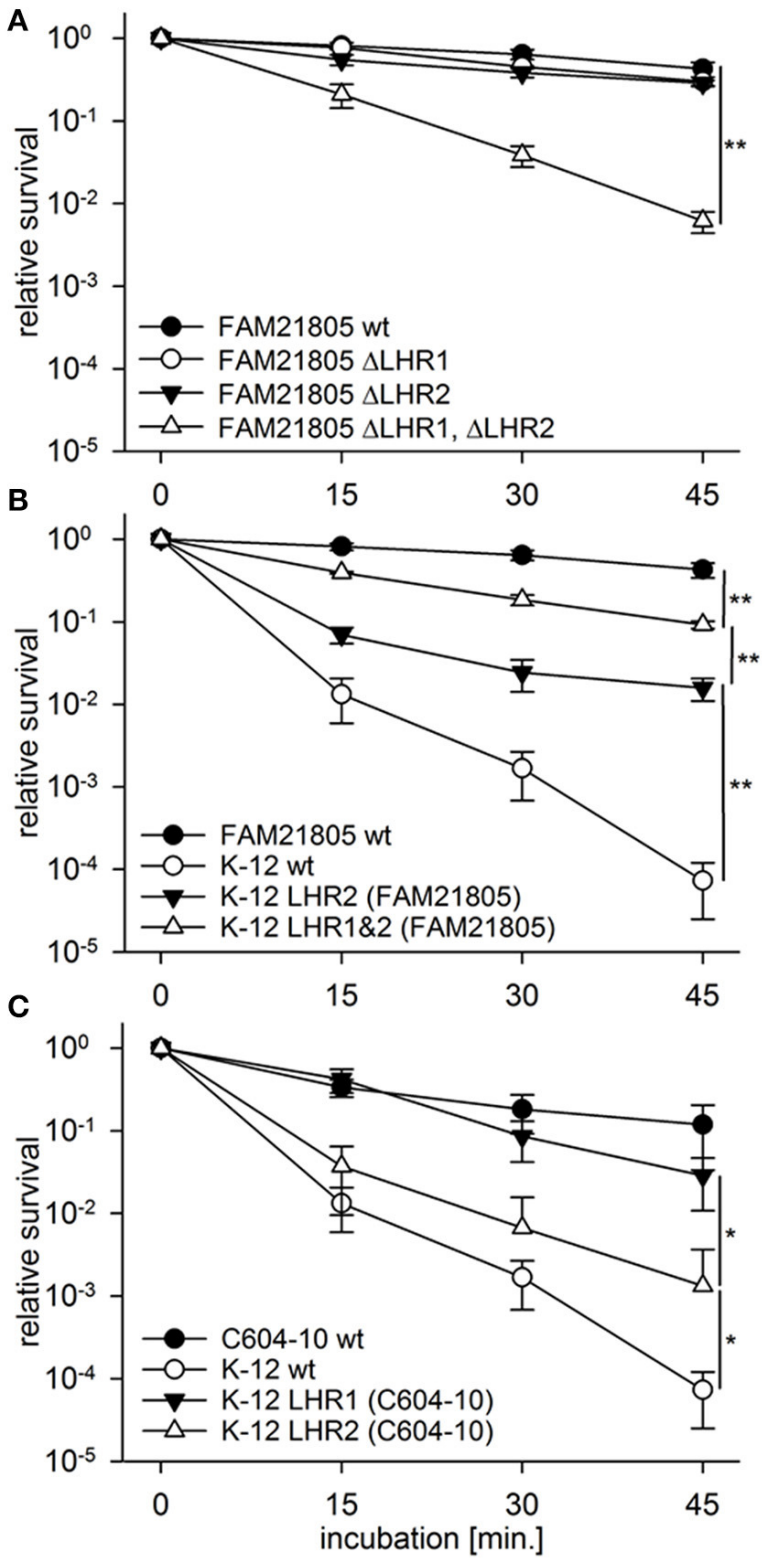
**Heat resistance assays of *E. coli* wild-type strains, FAM21805 mutants, and K-12 MG1655 transconjugants**. Relative survival compared to time point 0 of strains incubated at 55°C for 15, 30, and 45 min. **(A)** FAM21805 wild-type, -ΔLHR1, -ΔLHR2, and ΔLHR1ΔLHR2. **(B)** FAM21805 wild-type, K-12 MG1655 wild-type, and K-12 MG1655 transconjugants harboring LHR2 or both LHRs of FAM21805. **(C)** C604-10, K-12 MG1655 wild-type, and K-12 MG1655 transconjugants featuring LHR1 or LHR2 of C604-10. Error bars indicate standard deviations. Significances are indicated for the 45 min. time points based on one-tailed *t*-tests in **(A,B)**, and Mann-Whitney rank sum tests in **(C)**; ^*^p < 0.05, ^**^p < 0.01.

We have previously demonstrated that both LHRs in ESBL-producing *E. coli* isolate C604-10 confer heat resistance to this strain (Boll et al., 2016). To determine whether this was also the case with raw milk cheese *E. coli* isolate FAM21805, we generated a panel of LHR mutant strains with allelic replacements of designated regions with the *kan*^*r*^ gene (Table 1). For FAM21805 ΔLHR1, we deleted the region spanning from *orf2* (*sHsp20c)* to *orf16*. With regard to LHR2_FAM21805_, we deleted the region spanning from orf2 to the disrupted *orf14*, thereby excluding potential influence of removal of the inserted Microcin S system. As shown in Figure 4A, removal of either one of the LHRs did not affect survival of FAM21805 upon heat exposure. In contrast, survival of the FAM21805 double LHR mutant was severely reduced, thereby demonstrating that both LHRs of FAM21805 are functionally active and confer heat resistance.

We next sought to determine whether the LHRs of FAM21805 and C604-10 were amenable to horizontal gene transfer. We tagged LHR1 in both FAM21805 and C604-10 as well as LHR2_*C*604−10_ (all presumably chromosomally located) with genes encoding *kan*^*r*^ or *tet*^*r*^ by introducing them in the region immediately downstream of *orf16* (Table 1). Likewise, we inserted *kan*^*r*^ in a non-coding region on pFAM21805. We then carried out conjugative transfer assays using either one of the four LHR-tagged strains as donors and commensal *E. coli* strain K-12 MG1655 as recipient. Plate mating assays resulted in very high transfer frequencies for the tagged donor LHR2_FAM21805_ (7.56 ± 2.00 × 10^−1^ transconjugants per recipient). The donor with a tagged LHR2_*C*604−10_ produced transconjugants at very low levels. Standard plate matings with tagged LHR1 of both FAM21805 and C604-10 yielded no transconjugants at all. We proceeded to increase incubation time, spotting separate plate matings for each time point and incubating up to 12 days. This way, LHR1 transconjugants of both strains were generated and confirmed by PCR, albeit once again at very low levels. Notably, all of the LHR1_FAM21805_ transconjugants also appeared to have acquired pFAM21805. In contrast, we did not observe co-transfer of LHRs not selected for in any other case.

Confirming retained post-transfer functionality of the LHRs, all four types of K-12 MG1655 transconjugants exhibited significantly elevated survival upon heat exposure compared to the native strain (Figures 4B,C). The highest level of survival was observed in MG1655 carrying both LHR1_FAM21805_ and LHR2_FAM21805_ (Figure 4B), illustrating that both loci contribute to heat resistance in this bacterial background. In contrast, LHR2_*C*604−10_ appeared to more modestly confer heat protection. Finally, given the relative ease by which pFAM21805 was transferable to MG1655, we examined whether the plasmid could also be transferred to isolates belonging to diarrheagenic *E. coli* (DEC) and Shiga-toxin (Stx)-encoding *E. coli* (STEC) pathotypes. Through plate mating assays, we successfully transferred pFAM21805 tagged with antibiotic resistance cassettes to two STEC strains, FAM22873, and FAM23288, as well as two enteroaggregative *E. coli* (EAEC) strains, 55989 and the Stx-phage-cured German outbreak strain C227-11 φcu. As shown in Table 3, pFAM21805 conferred significantly elevated heat resistance to all four pathogenic *E. coli* strains. Strains conjugated with pFAM21805 ΔLHR2 exhibited the same levels of heat killing as did the non-conjugated strains, confirming that this effect was attributable to LHR2 (Table 3).

**Table 3:** **Heat resistance assays with pathogenic *E. coli* (EAEC and STEC) wild-type strains and the strains conjugated with pFAM21805 or with pFAM21805 ΔLHR2**.

**Strain**	**Relative survival^a^**		***p*-value^c^**
	**Average**	***SD*^b^**	
FAM22873 wild-type	5.64E-04	1.64E-04	–
FAM22873 ΔLHR2 (21805)	9.49E-04	1.08E-03	0.322
FAM22873 LHR2 (21805)	4.28E-02	2.24E-02	0.028
FAM23288 wild-type	9.03E-04	2.04E-04	–
FAM23288 ΔLHR2 (21805)	4.79E-04	5.38E-04	0.124
FAM23288 LHR2 (21805)	1.02E-02	8.12E-03	0.029
55989 wild-type	3.60E-04	1.54E-04	–
55989 ΔLHR2 (21805)	5.05E-04	1.98E-04	–
55989 LHR2 (21805)	3.91E-01	1.23E-01	0.007
C227-11 φcu	3.23E-04	4.89E-04	–
C227-11 φcuΔ LHR2 (21805)	1.20E-04	1.08E-04	–
C227-11 φcu LHR2 (21805)	4.27E-01	1.39E-01	0.001

a *Relative survival at time point 45 min. compared to 0 min. incubation at 55°C*.

b *SD, standard deviation*.

c *p-value of direct comparison (one-tailed t-test or Mann-Whitney rank sum) against corresponding wild-type strain*.

### Screening for other locus of heat resistance-related stress response phenotypes

We next sought to determine whether the LHRs confer other stress-related advantages to their bacterial host in addition to enhanced thermotolerance. Since homologs of most of the ORFs are present in both of the LHR, we focused on the combined effect of removing both loci in FAM21805.

We first looked at the ability of FAM21805 wild-type and the ΔLHR1ΔLHR2 mutant to survive oxidative stress. However, neither killing nor growth challenge assays with hydrogen peroxide revealed any significant differences between the wild-type and mutant strain (data not shown). Thus, there seems to be no clear protective effect of either LHR against the oxidative action of H_2_O_2_.

To examine the potential effect of osmolarity and pH, we next screened the wild-type and ΔLHR1ΔLHR2 mutant strain using phenotypic microarrays (PM) 9 and 10 from the Biolog system, which measures activity of the bacterial metabolism via respiratory action (reduction of a redox dye). A biological triplicate with both PM 9 and 10 revealed only one consistent phenotypical difference between the two strains. PM9 contains four wells challenging bacteria with sodium benzoate at concentrations of 20, 50, 100, and 200 mM (each at pH 5.2). At a concentration of 100 mM, the wild-type strain consistently started respiring after ~36 h of incubation, while the LHR double mutant was unable to do so. Both strains were able to respire at 20 and 50 mM sodium benzoate while both were unable to do so at 200 mM over the entire 72 h incubation. FAM21805 ΔLHR1&2 complemented with either LHR2 or LHR1&2 were able to respire at 100 mM sodium benzoate (pH 5.2) after ~36 h, like the wild-type (single replicate). Notably, K-12 MG1655 transconjugants with LHR2 or LHR1&2 of FAM21805 did not show this sodium benzoate related phenotype (biological duplicate).

### The pFAM21805 plasmid increases biofilm formation of *E. coli* MG1655

**Figure 5:**
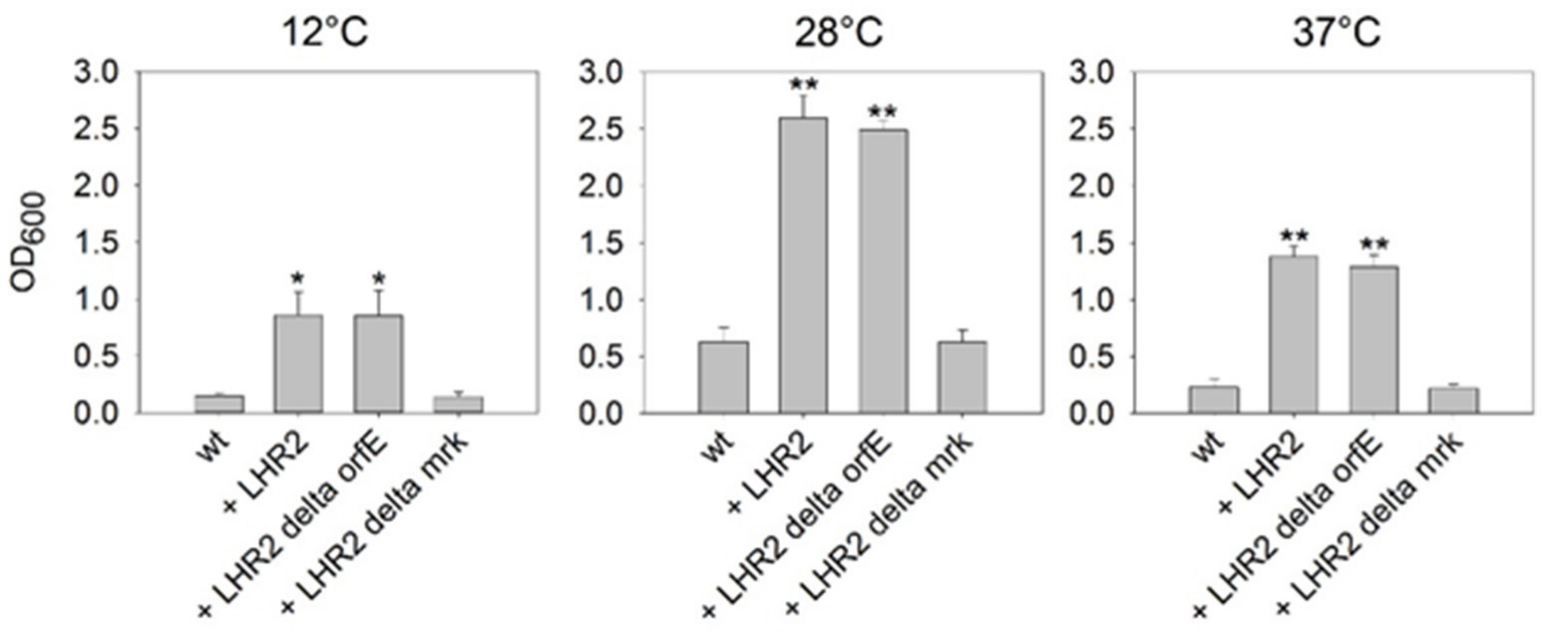
**Impact of pFAM21805 on biofilm formation of K-12 MG1655**. Biofilm formation of *E. coli* K-12 MG1655 wild-type and K-12 MG1655 transconjugants harboring pFAM21805, pFAM21805 Δ*orfE* (deletion of LHR2-encoded putative di-guanyl cyclase), or pFAM21805 Δ*mrk* (deletion of type 3 fimbriae genes). Bacteria were grown in ABTCAA media for 48 h at 12, 28, and 37°C, after which biofilm formation was quantified by crystal violet (CV) staining. Bars represent averages and standard deviations (^*^p < 0.05, ^**^p < 0.001, tested by 1-way ANOVA vs. K-12 wt control, α = 0.05, Holm-Sidak).

Biofilm formation is an important contributor to persistence of bacteria in both food processing and clinical settings (Abdallah et al., 2014). A combination of biofilm formation with heat resistance would increase a strain's persistence even further (Bojer et al., 2011). As described above, pFAM21805 contains the *mrk* gene cluster encoding type 3 fimbriae, which are strongly associated with bacterial biofilm formation (Burmølle et al., 2008; Hufnagel et al., 2015). Moreover, *orfE* of LHR2_FAM21805_ encodes a putative di-guanyl cyclase, and c-di-GMP signaling has been shown to affect biofilm formation (Burmølle et al., 2008; Schroll et al., 2010; Hufnagel et al., 2015). Thus, both factors could potentially contribute to increased biofilm formation in *E. coli*. To investigate if this was the case, we replaced either *mrkABCDF* or *orfE* in FAM21805 with *tet*^*r*^ (Table 1), and transferred pFAM21805 from the two corresponding mutant strains to K-12 MG1655. We then performed crystal violet (CV) assays with K-12 MG1655 *nal*^*r*^ *rif*^*r*^ and its transconjugants in ABTCAA for 48 h at 12, 28, and 37°C. As shown in Figure 5, the presence of intact pFAM21805 significantly increased biofilm production of MG1655. Moreover, our results show that the *mrk* locus—but not the putative LHR2-encoded di-guanyl cyclase—was required to increase biofilm formation in K-12 MG1655 transconjugants. Thus, pFAM21805 has the potential to confer both enhanced survival during heat stress and adhesive properties at a wide range of temperatures to *E. coli* recipient strains.

## Discussion

Heat treatment is a commonly used process in the food industry and the main technology to reduce bacterial load. It is therefore crucially important to understand the mechanisms mediating increased heat resistance in potentially pathogenic bacteria as well as its origin, potential for distribution, and possible cross-protective effects associated with this phenotype.

We and others have recently described the LHR, which is present in and confers heat resistance to a variety of *Enterobacteriaceae* as well as *Pseudomonas* spp. (Bojer et al., 2010; Lee et al., 2015; Mercer et al., 2015; Boll et al., 2016). In contrast to *K. pneumoniae*, where the LHR appears to predominantly be located on plasmids, thus far, only chromosomally located LHRs have been reported in *E. coli*, including the fully sequenced strain P12b (Bojer et al., 2010; Liu et al., 2012; Mercer et al., 2015). This likely explains the much lower overall predicted prevalence of LHR in *E. coli* (~2%) compared to *K. pneumoniae* (1/3 of clinical isolates) (Bojer et al., 2010; Mercer et al., 2015). However, when screening for LHR marker genes in a collection of *E. coli* isolates from dairy production, we observed a much higher fraction of LHR-positive isolates (~36%). This most likely reflects a selection process in which LHR-positive isolates survive the thermal processing steps to a much greater extent than LHR-negative isolates (Marti et al., 2016). Further studies are needed to evaluate the occurrence of similar selection processes under sub-pasteurization heat treatments in clinical usage and food production.

In this study, we were able to more closely characterize a subset (90 isolates) of the collection of *E. coli* dairy isolates with regard to the presence of LHR. While distinct variants of LHRs exist in different species, the *clpK* gene (as well as the upstream located *sHsp20c*) are always present. We therefore used PCRs specifically targeting *clpK1* and *clpK2* as markers for *E. coli* LHR1 (Mercer et al., 2015) and the newly discovered variant LHR2, found in the ESBL-encoding *E. coli* isolate C604-10 (Boll et al., 2016), respectively. We observed a strong correlation between the presence of LHR and a heat resistant phenotype, with all of the heat resistant isolates harboring LHR1, with the exception of FAM22891, which tested *clpK2* single positive. Nearly half of the LHR1-positive isolates additionally also harbored LHR2, and these isolates exhibited the highest levels of heat resistance overall. These findings stress the need for screening for both LHR1 and LHR2 to detect highly heat resistant isolates and validate the use of primers targeting the *clpK* genes as markers for the LHRs.

We also examined whether the LHRs would be amenable to horizontal gene transfer. Focusing on C604-10 and *E. coli* raw milk cheese isolate FAM21805, both of which harbor both LHR1 and LHR2, we indeed found that all four LHRs were transferrable to *E. coli* K-12; however, with very different rates of transfer. Interestingly, compared to the other three LHRs, LHR2_FAM21805_ was transferred at a much higher rate. MinIon-based sequencing revealed that, unlike the other LHRs, LHR2_FAM21805_ was encoded on a large IncFII-type plasmid, pFAM21805. This plasmid also contained a *tra* operon (Lawley et al., 2003), thus explaining why this plasmid was so readily transferred by conjugation. Importantly, pFAM21805 was also readily conjugated into STEC and EAEC, both of which are pathotypes associated with foodborne outbreaks (Rasko et al., 2011; Farrokh et al., 2013; Robertson et al., 2016). While thus far, no studies have described the natural presence of LHR in these diarrheal pathotypes, this finding highlights the potential for such cases to occur.

The fact that LHR1 was transferred at a much lower rate than the plasmid-encoded LHR2 from FAM21805 suggests that LHR1 is located either on the chromosome or on a non-conjugative plasmid in this strain. Gel electrophoresis of the LHR1 K-12 MG1655 transconjugant (also harboring pFAM21805) demonstrated the presence of a single plasmid identical in size to that of the pFAM21805 K-12 MG1655 transconjugant (data not shown). This rules out the location of LHR1 on the second large plasmid present in FAM21805. We instead aligned Illumina sequences from the LHR1 K-12 MG1655 transconjugant with the genome sequence of MG1655. Based on this, we identified 1,708 SNPs within a region spanning ~700 kb of the MG1655 genome. The same high SNP frequency occurred across the entire MG1655 genome following alignment with Illumina sequences from FAM21805. Taking together, these findings suggest that LHR1_FAM21805_ was transferred horizontally to MG1655 as part of a 700 kb region of FAM21805. While the underlying mechanism for this phenomenon remains to be determined, the LHR-containing DNA region could have been transferred to FAM21805 by conjugal mating with a high frequency recombinant (Hfr) donor strain with a conjugative plasmid integrated into its genome (O'Gorman et al., 1996). A similar scenario describing a chimeric *K. pneumoniae* strain having taken up a large portion of genomic DNA from another strain has previously been described (Struve et al., 2015). In any case, such a transfer mechanism would expectedly occur at a very low frequency, co-inciding with very low transfer frequency of LHR1_FAM21805_ in our study.

In addition to LHR2, pFAM21805 also contained the *mrkABCDF* locus encoding type 3 fimbriae, which are considered a major virulence factor of *K. pneumoniae* allowing the organism to produce extensive biofilm (Schroll et al., 2010; Andrade et al., 2014). In K-12 MG1655, conjugation with pFAM21805 lead to significantly increased biofilm production at 12, 28, and 37°C, which may in turn increase frequency of horizontal gene transfer (Burmølle et al., 2014; Rossi et al., 2014). The fact that the entire range of temperatures tested saw an increase in biofilm formation suggests potential beneficial effects for a host of this plasmid both in the environment and *in vivo*.

Moreover, the plasmid also contained three bacteriocins (along with their respective self-immunity genes): Colicin B, Colicin M, and Microcin S. Although the functionality of the bacteriocins remains to be verified, having the ability to produce multiple bacteriocins most likely provides the bacterial host of the plasmid with an expanded killing range, and thus a competitive advantage in multispecies communities (Gordon and O'Brien, 2006).

The pFAM21805 plasmid did not encode any antimicrobial resistance genes. This is in contrast to *K. pneumoniae*, where the LHR is often present on plasmids harboring resistances to tetracycline, neomycin, trimethoprim, sulfamethoxazole as well as encoding ESBL genes such as CTX-M-15, which confers resistance to third-generation cephalosporins (Bojer et al., 2012). Importantly, FAM21805 has been shown to be able to harbor ESBL-encoding conjugative plasmids and is able to act as donor of these (Marti et al., 2016), and the *tra* operon on pFAM21805 could likely enable conjugation of mobilizable (resistance) plasmids in other hosts of this LHR2 plasmid.

Interestingly, we observed a strong correlation between the presence of the *mrk* gene cluster and LHR in our collection of dairy *E. coli* isolates (all 13 *mrk* positive isolates also harbored *clpK1* and some also *clpK2*). In light of this, we performed whole-genome sequencing on two *mrk*- and *clpK2*-positive isolates but found that neither isolate harbored pFAM21805 or a highly similar plasmid. Thus, in spite of the fact that the plasmid was readily transferred and provided clear benefits to its host, it did not appear to be widespread among these isolates. However, this does not rule out the possibility that other LHR-encoding plasmids exist, some of which may potentially harbor LHR1-like heat resistance clusters and *mrk*, similar to pFAM21805. Notably, thus far in *E. coli*, the *mrk* gene cluster has only been reported as located on conjugative plasmids (Burmølle et al., 2008), which is in agreement with our finding of *mrk* on pFAM21805. Further studies are needed to clarify the role of the *mrk* gene cluster and biofilm production capacity in *E. coli* isolates being persistent in hospitals or food-production.

The exact function of many of the ORFs of the LHRs remains to be unraveled. However, the fact that the majority of them are highly conserved strongly suggests that they all play a beneficial role for their host. Notably, some of them are predicted to act as ion-exchangers or proteases/peptidases suggesting that they may be involved in handling osmotic- or heat stress (Mercer et al., 2015). The ClpK chaperone itself shares many structural properties with ClpB, which is known to play a critical role in survival following various types of stress (Squires et al., 1991; Ekaza et al., 2001; Lourdault et al., 2011). We therefore endeavored to identify other stress response phenotypes associated with LHRs in FAM21805. However, both H_2_O_2_ growth challenge and killing assays revealed no significant differences between the FAM21805 wildtype and LHR double mutant strain in response to this stressor. Assays with the phenotypic microarrays 9 and 10 of the Biolog system did reveal one phenotype: The FAM21805 wildtype was able to respire in 100 mM sodium benzoate (pH 5.2) after 36 h, while its LHR1&2 double mutant could not. This phenotype could be complemented in FAM21805 ΔLHR1&2 with either LHR2 or LHR1&2, but not transferred to K-12 MG1655 by conjugation. The phenotype appears to be dependent on the genomic background around LHR2. Benzoate is being used as a preservative in a range of foods. Although a difference was only observed in one scenario here, it indicates that there could be non-thermal stresses where LHR would confer an advantage to the host isolate.

In conclusion, we have characterized in detail the presence of a recently discovered variant of LHR (LHR2) and demonstrated its presence on a plasmid in the highly heat resistant dairy *E. coli* isolate FAM21805. This plasmid was transferable at much higher rates than the presumably chromosomal LHRs tested, and conferred LHR2-dependent heat resistance as well as *mrk*-dependent biofilm formation capabilities to recipient *E. coli*, including pathogenic strains. In addition, the plasmid also harbored three bacteriocins and corresponding self-immunity proteins. Selection for and acquisition of this “survival” plasmid by pathogenic organisms, e.g., in food production environments, may pose great concern and emphasizes the need to screen for the presence of LHR genes in isolates.

## Author contributions

EB and RM designed the work, collected, analyzed and interpreted the data and drafted the article. HH, SO, and MS analyzed and interpreted the data and critically revised the article. KN analyzed and interpreted the data. SK critically revised the article. KK and JH interpreted the data and critically revised the article. CS designed the work, interpreted the data and critically revised the article.

### Conflict of interest statement

The authors declare that the research was conducted in the absence of any commercial or financial relationships that could be construed as a potential conflict of interest.
