# Supplementary material for: Turn Up the Heat—Food and Clinical Escherichia coli Isolates Feature Two Transferrable Loci of Heat Resistance
Source: Front Microbiol. 2017 Apr 7;8:579. doi: 10.3389/fmicb.2017.00579 (PMC5383660; doi:10.3389/fmicb.2017.00579)
Supplement: Supplementary file 1 [file Table1.PDF]

Suppl. Table 1. Predicted proteins encoded by LHR2 of *E. coli* FAM21805 and C604-10.

**ORF**	**# of amino acids**	**Protein product (Conserved Domains)**	**Homolog in $LHR1_{FAM21805}$**	**Homolog identity (amino acids)**
1 [1]	93	DNA binding protein (HTH)	Yes	74%
2	189	Small heat shock protein, sHsp20c	Yes	86%
3	973	Clp ATPase, ClpK	Yes	95%
A [2]	513	Cardiolipin synthetase (Cls)	Partial	-
B	609	ATP-dependent metalloprotease (FtsH)	Yes	90%
7	152	Small heat shock protein (ACD)	Yes	97%
8	302	Hypothetical protein (YfdX)	Yes	88%
9	295	Hypothetical protein (YfdX)	Yes	96%
10	203	Hypothetical membrane protein (HdeD)	Yes	98%
C	303	Mechanosensitive ion channel (MscS)	No	-
D	230	Hypothetical protein	No	-
12	146	Thioredoxin (TRX)	Yes	91%
13	568	Sodium/hydrogen exxhanger (KefB, TrkA_N)	Yes	76%
E	238	Diguanylate cyclase (PAS, GGDEF)	No	-
15	320	Zn-dependent protease (M48)	Yes	76%
16	387	Trypsin-like serine protease (Tryp_SPc, PDZ)	Yes	94%

[1] *orf1* of $LHR1_{FAM21805}$ is absent in $LHR2_{FAM21805}$ but present in $LHR2_{C604-10}$. See **Fig. 1**.

[2] Remnants of the N-terminal region (76aa) and C-terminal region (23aa) of *orfA* are present in $LHR1_{FAM21805}$. See **Fig. 1**.
