# Supplementary material for: Turn Up the Heat—Food and Clinical Escherichia coli Isolates Feature Two Transferrable Loci of Heat Resistance
Source: Front Microbiol. 2017 Apr 7;8:579. doi: 10.3389/fmicb.2017.00579 (PMC5383660; doi:10.3389/fmicb.2017.00579)
Supplement: Supplementary file 2 [file Image1.pdf]

Supplementary Figure 1

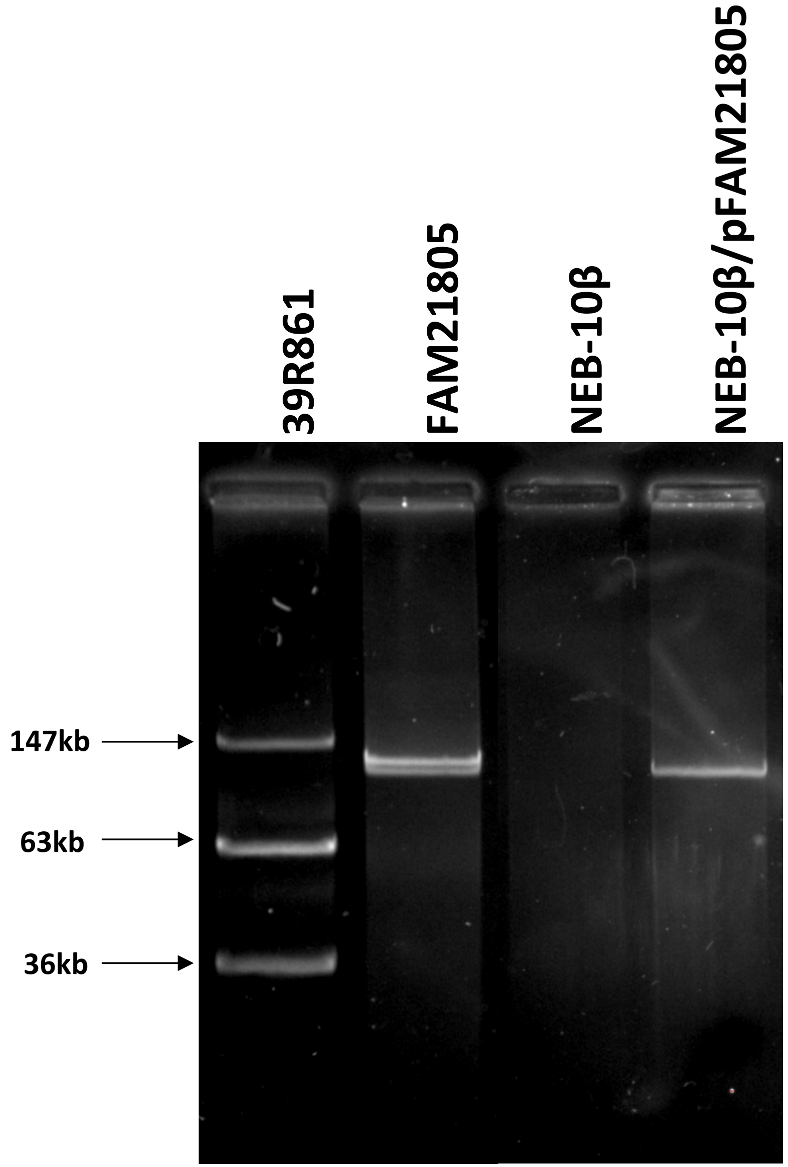

Suppl. Fig. 1. Gel electrophoresis of plasmids purified from *E. coli* strain 39R861 (reference with plasmids of known depicted sizes), FAM21805 (two plasmids), laboratory *E. coli* strain NEB-10β and NEB-10β with pFAM21805 inserted by electroporation.
